# A Review of Acute and Long-Term Neurological Complications Following Haematopoietic Stem Cell Transplant for Paediatric Acute Lymphoblastic Leukaemia

**DOI:** 10.3389/fped.2021.774853

**Published:** 2021-12-23

**Authors:** Melissa Gabriel, Bianca A. W. Hoeben, Hilde Hylland Uhlving, Olga Zajac-Spychala, Anita Lawitschka, Dorine Bresters, Marianne Ifversen

**Affiliations:** ^1^Cancer Centre for Children, The Children's Hospital at Westmead, Sydney, NSW, Australia; ^2^Department of Radiation Oncology, University Medical Center Utrecht, Utrecht, Netherlands; ^3^Princess Máxima Center for Pediatric Oncology, Utrecht, Netherlands; ^4^Department of Pediatrics and Adolescent Medicine, Copenhagen University Hospital Rigshospitalet, Copenhagen, Denmark; ^5^Department of Pediatric Oncology, Hematology and Transplantology, Poznan University of Medical Sciences, Poznań, Poland; ^6^Haematopoietic Stem Cell Transplant Unit, St. Anna Children's Hospital, Medical University Vienna, Vienna, Austria

**Keywords:** haematopoietic stem cell transplant, neurotoxicity, neurological complications, paediatric, acute lymphoblastic leukaemia

## Abstract

Despite advances in haematopoietic stem cell transplant (HSCT) techniques, the risk of serious side effects and complications still exists. Neurological complications, both acute and long term, are common following HSCT and contribute to significant morbidity and mortality. The aetiology of neurotoxicity includes infections and a wide variety of non-infectious causes such as drug toxicities, metabolic abnormalities, irradiation, vascular and immunologic events and the leukaemia itself. The majority of the literature on this subject is focussed on adults. The impact of the combination of neurotoxic drugs given before and during HSCT, radiotherapy and neurological complications on the developing and vulnerable paediatric and adolescent brain remains unclear. Moreover, the age-related sensitivity of the nervous system to toxic insults is still being investigated. In this article, we review current evidence regarding neurotoxicity following HSCT for acute lymphoblastic leukaemia in childhood. We focus on acute and long-term impacts. Understanding the aetiology and long-term sequelae of neurological complications in children is particularly important in the current era of immunotherapy for acute lymphoblastic leukaemia (such as chimeric antigen receptor T cells and bi-specific T-cell engager antibodies), which have well-known and common neurological side effects and may represent a future treatment modality for at least a fraction of HSCT-recipients.

## Introduction

Neurological complications occurring post paediatric haematopoietic stem cell transplantation (HSCT) contribute significantly to morbidity and mortality both in the short and long term. The incidence of neurotoxicity in children following HSCT, for a variety of indications, ranges in the literature from 11–59% (1–6). There is a paucity of literature examining neurological complications specifically in children undergoing HSCT for ALL.

Moreover, a widely cited post-mortem study showed that 90% of the 180 HSCT recipients (adults and children; age range 1–48 years) had evidence of central nervous system (CNS) abnormality and that this was the cause of death in 17% (7). A number of studies have shown that the outcomes of HSCT are poorer for patients who develop acute neurotoxicity (8, 9).

The majority of paediatric studies of the neurological effects of HSCT have focussed on acute neurotoxicity (1–6). As more children undergoing HSCT for acute lymphoblastic leukaemia (ALL) become long-term survivors, we are obligated to understand the long-term neurological consequences of this treatment modality. With this in mind, the current review explores both acute and long-term neurological complications occurring after HSCT in paediatric patients with ALL.

Converging evidence from related fields (hypoxic and traumatic brain injury, radiotherapy and ALL therapy not including HSCT) has identified the vulnerability of the paediatric brain to injury (10–12). This collective evidence strongly suggests that radiotherapy, and possibly chemotherapy also, added to neurological injury occurring as a result of acute central nervous system (CNS) complications may have profound effects on brain maturation and consequently on cognitive function; indeed, fatigue and low mood have been shown to be associated with neurocognitive deficits post cancer therapy and HSCT (13–17). Because of the demonstrated close relationship between neurocognitive deficits and fatigue, with subsequent impacts upon educational and vocational outcomes and quality of life, we have elected to review these together under the term “long-term neurocognitive impacts” of HSCT.

We aim to provide a comprehensive review of both the acute and long-term neurological complications observed in children who have undergone HSCT for ALL. Although a little outside the scope of a HSCT review, we have also chosen to include a brief review of the neurotoxicity associated with CAR T cell therapy. We think it is important as CD19-Chimeric antigen receptor (CAR) T cell therapy becomes more widely available for children with relapsed or refractory CD19+ B ALL, including post HSCT relapse. Table 1 outlines the risk factors associated with the neurotoxicities reviewed in this paper.

**Table 1:** Risk factors for acute and late neurotoxicity effects after allogeneic HSCT for pediatric ALL and CD19+ CAR T cell therapy.

**Acute neurotoxicity**	**Risk factors**
Infections - Viral - Fungal - Bacterial - Toxoplasmosis	Pre-transplant viral status; EBV, CMV, HSV, VZV, HHV6, (JCV) Toxoplasmosis GvHD Immunosuppression
Drug neurotoxicity; Posterior reversible encephalopathy syndrome (PRES), acute toxic leukoencephalopathy (ATL), leukencephalopathy, seizures, peripheral neuropathy, headaches, hallucinations, somnolence, cranial nerve palsies, weakness - Fludarabine - Busulfan - Nelarabine	Fludarabine Busulfan Nelarabine Vincristine Previous CNS disease Advanced disease status Older age
Posterior reversible encephalopathy syndrome (PRES)	Calcineurin inhibitors Sirolimus Everolimus Dexamethasone Fludarabine Hypomagnesaemia Umbilical cord stem cell source G-CSF
CNS GvHD; cerebrovascular disease, demyelinating disease, immune-mediated encephalitis	Acute and chronic GvHD autoimmunity
Immune effector cell-associated neurotoxicity syndrome (ICANS)	High disease burden pre CAR-T cells High peak CAR T cell expansion in blood Extramedullary disease Younger age Pre-existing neurological abnormalities High CAR T cell dose Cytopaenias High grade cytokine release syndrome (CRS)
**Long-term neurotoxicity**	**Risk factors**
Cerebrovascular accident (CVA)	Cranial irradiation TBI-based conditioning regimen Cardiovascular risk profile Metabolic syndrome Disease status at HSCT (>CR1) ≥2 transplants
Secondary CNS malignancy	Cranial irradiation TBI-based conditioning regimen CNS leukaemia before HSCT Young age (<6 years old at HSCT, <3 higher risk) Unrelated donor stem cell source NF-1 Chronic GvHD/immunosuppression
Peripheral neuropathy	Vincristine Nelarabine Chronic GvHD Immunosuppressive drugs (cyclosporine, tacrolimus)
Neurocognitive effects	Cranial radiotherapy TBI Young age (<3–4 years old at HSCT) Methotrexate Other CNS prophylaxis therapy Low socioeconomic status Low pre-HSCT neurocognitive functioning
Fatigue	Chemotherapy Cranial radiotherapy Medical comorbidities Immunosuppression Psycho-social status Reduced physical activity
Decreased HRQoL	TBI Chronic health conditions after HSCT Chronic pain Anxiety Fatigue Unemployment/sick leave Reduced physical activity

*ATL, acute toxic leukoencephalopathy; CAR-T, chimeric antigen receptor T-cell; CMV, cytomegalovirus; CNS, central nervous system; CVA, cerebrovascular accident; EBV, Epstein-Barr virus; G-CSF, granulocyte colony-stimulating factor; GvHD, graft versus host disease; HHV, human herpes virus; HRQoL, health-related quality of life; HSV, herpes simplex virus; ICANS, immune effector cell-associated neurotoxicity syndrome; JCV, JC polyomavirus; NF-1, neurofibromatosis type 1; PRES, posterior reversible encephalopathy syndrome; TBI, total body irradiation; VZV, varicella zoster virus*.

Acute neurological complications are broadly divided into infectious or non-infectious causes. Acute non-infectious neurotoxicity relates primarily to drug toxicity. We review here the neurotoxicity of common chemotherapy drugs (busulfan and fludarabine) used for non-TBI-based conditioning predominantly in younger children with ALL. We have included the neurotoxicity of nelarabine, which is often used as a bridge to transplant for patients with relapsed or refractory T-cell ALL. We review the risk of posterior reversible encephalopathy, which is most commonly associated with calcineurin inhibitors used for graft-vs.-host disease (GvHD) prophylaxis in the majority of patients undergoing HSCT. Lastly, in the acute section, we review GvHD-associated CNS disease. The long-term complications we review are: secondary CNS tumours, peripheral neuropathy, ischaemic complications and neurocognitive impacts (including cognition, fatigue and quality of life).

## Acute Neurotoxicity Post HSCT

### Infectious Causes of Acute Neurotoxicity

Infections due to viruses, bacteria, fungi and parasites are the leading cause (35%) of acute neurotoxicity in paediatric ALL patients who have undergone HSCT. Clinical infectious manifestations may be absent in transplant recipients due to host immunosuppression, but CNS infection should be suspected upon occurrence of new neurological symptoms, fever or other systemic infection, especially in the early post-transplant period (18).

Pre-transplant viral status—defined as a higher number of recipients who are seropositive to the herpes groups—correlates with the risk of neurologic complications post-transplant (19, 20). Following recovery from primary infection, human herpes viruses (HHVs) enter a state of latency in lymphocytes and monocytes/macrophages. Thus, viral CNS infections are frequently caused by reactivation of these viruses including herpes simplex virus, Epstein-Barr virus, varicella zoster virus, cytomegalovirus (CMV), HHV-6 and human polyomavirus (also known as JC polyomavirus or JCV). Approximately 40% of HSCT recipients experience early reactivation of herpes viruses after transplantation, especially those suffering from GvHD (21, 22). Appropriate prophylaxis matched to the herpes group serological status of donor and recipient may protect from reactivation of these infections.

Another important cause of infectious CNS morbidity in paediatric ALL patients who have had an allogeneic HSCT is invasive opportunistic fungal disease (23). The predominant causal fungal genus is *Aspergillus*, with *Aspergillus fumigatus* prevailing over other species. Since *Aspergillus* is rarely recovered from blood cultures, the diagnosis of proven invasive opportunistic fungal disease may require invasive procedures to obtain tissue; however, such procedures are fraught with risks of morbidity or mortality in this patient population, especially when involving the CNS. Thus, while histopathological diagnostic tools will always remain important to pursue a specific definitive diagnosis, non-invasive diagnostic tools have largely replaced tissue diagnosis of invasive opportunistic fungal diseases in paediatric HSCT recipients. Imaging with computed tomography (CT) or magnetic resonance imaging (MRI) scans, serological testing including the serum galactomannan assay for Aspergillus and the serum (1,3)-β-d-glucan (BDG) antigen test, and molecular techniques including polymerase chain reaction-based assays with higher specificity and sensitivity than serological assessment can identify and lead to earlier treatment (24).

Toxoplasmosis is an opportunistic infection caused by the parasite *Toxoplasma gondii*. Infection in an immunocompetent host leads to latency of the parasite as cysts in various organs. *Toxoplasma gondii* allograft transmission or reactivation of latent infection may be present in HSCT patients, especially in countries where toxoplasmosis is more prevalent (25, 26). Toxoplasmosis in patients following HSCT frequently involves the CNS, both as an isolated cerebral infection or as disseminated disease. The typical MRI features include multiple lesions in the subcortical white matter, basal ganglia, and cerebellum, with focal nodular or rim enhancement present in some lesions (27). Mortality rate in cerebral toxoplasmosis is very high. The incidence is reduced by use of trimethoprim-sulfamethoxazole prophylaxis in recipients with positive serology or a seropositive donor (18).

Brain abscess is a rare but severe CNS complication of HSCT. Similar to *Aspergillus* and *Toxoplasma* infections, bacterial abscesses may not show significant enhancement because the imaging characteristics of cerebral infections relate to the immune status of the HSCT recipient. With brain abscesses, encapsulation around the abscess cavity—which indicates the occurrence of sequential events involving neovascularization, inflammatory cell migration, and immune response—is not usually complete, and a mass effect or oedema around the lesion caused by an inflammatory infiltrate of polymorphonuclear cells is relatively rare (27).

Current diagnostic techniques for suspected infectious cases rely on prior knowledge of the likely causative agent. Informed by clinical presentation, epidemiological data, guidelines and local resources, a laboratory will perform targeted tests for a disease. These are largely confined to specific PCR or serological assays. This approach has fundamental limitations, and contributes to the relatively high proportion of encephalitis cases that remain undiagnosed. Thus, there is a need for improved diagnostic methods for encephalitis. A method which has recently been applied to pathogen detection in cases of encephalitis is metagenomics analysis using next generation sequencing (NGS). NGS has striking potential to identify undiagnosed pathogens and thus reduce the number of cases with unknown aetiology. It also has utility for pathogen detection in other clinical syndromes, such as respiratory infections, therefore the implementation of this technique in clinical laboratories would have wider implications for diagnosis of infection beyond encephalitis (28).

### Non-infectious Causes of Acute Neurotoxicity

Non-infectious aetiology of acute neurotoxicity largely relates to drug toxicities, vascular events, and metabolic and immune-mediated (CNS GvHD) causes. We now describe each of these in turn.

#### Drug Toxicities

The drugs used for conditioning prior to HSCT (e.g., fludarabine and busulfan) and for GvHD prophylaxis can cause toxic leukoencephalopathy.

##### Fludarabine

Neurotoxic side effects of fludarabine when used in the treatment of haematological malignancies have long and widely been described (29–42). Studies have shown that neurological complications usually occur 20–250 days post HSCT and present with a variety of clinical manifestations including visual disturbances, blindness, weakness, encephalopathy and coma. Risk factors include higher doses of fludarabine, advanced disease status, older age and renal impairment. In studies where MRI was performed, there was evidence of toxic leukoencephalopathy with either focal or widespread changes consistent with white matter demyelination.

Beitinjaneh et al. evaluated toxic encephalopathy in 1,597 recipients (both adults and children) following fludarabine-based conditioning prior to HSCT for a variety of indications (43). The incidence of severe leukoencephalopathy was 2.4%. They described three distinct clinical syndromes with associated MRI changes:

1. Posterior reversible encephalopathy syndrome (PRES) which presented with headache, visual disturbance and seizures. MRI demonstrated subcortical and cortical white matter changes.
2. Acute toxic leukoencephalopathy (ATL) which was associated with cognitive impairment, visual disturbance and decreased levels of consciousness. MRI changes were seen in the deep white matter.
3. Other leukoencephalopathy which was clinically similar to ATL but had less significant deep white matter changes on MRI.

The authors found it difficult to discern calcineurin inhibitor-associated PRES from fludarabine-associated PRES (43). Those with ATL had a worse prognosis than those with PRES Risk factors for fludarabine-associated leukoencephalopathy were older age, renal impairment, fludarabine dose, a previous fludarabine-based HSCT and previously treated CNS disease.

In a recent study of 29 adults undergoing HSCT for high risk haematological malignancies by Bethge et al., fludarabine 200 mg/m^2^ was initially used but needed to be reduced to 160 mg/m^2^ after four patients developed severe neurotoxicity. This study used haploidentical donors with CD3^+^/CD19^+^ depletion of the stem cell product and mycophenolate mofetil as GvHD prophylaxis. Calcineurin inhibitors were not used in this study, so the neurotoxicity was attributed to fludarabine alone.

##### Busulfan

Busulfan-based chemoconditioning is used as an alternative to TBI for younger patients undergoing HSCT for ALL. Seizures are the most common neurological side effect associated with busulfan. The drug has good penetration into the cerebrospinal fluid (CSF), with levels similar to that in the plasma (44). The risk of seizures appears to be dose dependent and related to high drug concentrations in the CNS (44–46). In early studies (which included adults and children undergoing HSCT for a variety of indications), in which patients would not have received prophylaxis for seizures, the incidence of seizures was in the order of 10% (44, 47, 48). The use of anticonvulsant prophylaxis with busulfan is now the standard of care for paediatric patients and a variety of drugs are used (49–51). The risk of seizures has been ameliorated by the routine use of prophylactic anticonvulsants and targeted busulfan pharmacokinetics (52).

##### Nelarabine

Nelarabine is used for the treatment of relapsed or refractory T-cell ALL (53–58). We are including nelarabine in this review as it is often used as a bridge to HSCT in these patients (57). It is associated with significant neurotoxicity with up to one-third of children reported to develop severe peripheral sensory or motor neuropathy or grade 3 or 4 central neurotoxicity (seizures, headaches, hallucinations, somnolence, weakness and cranial nerve palsies) when treated with nelarabine (53, 59–61). The incidence of neurotoxicity appears to be the same when nelarabine is combined with other chemotherapeutic agents (58, 62–64). The majority of neurological side effects appear to be gradually reversible but some can persist in some children (53, 58, 64). As the number of patients reported to have nelarabine-associated neurotoxicity in the literature is relatively small, the impact of this neurotoxicity on potential HSCT neurological complications is unclear. Therefore, we suggest it is important for physicians conducting HSCT to monitor patients who have experienced nelarabine neurotoxicity more closely than they otherwise would.

#### Posterior Reversible Encephalopathy Syndrome

Calcineurin inhibitors such as cyclosporine and tacrolimus form the backbone of GvHD prophylaxis for allogeneic HSCT in both children and adults. PRES post HSCT is most commonly associated with these agents. PRES has also been reported with sirolimus, everolimus and dexamethasone use (65–67). Other risk factors for PRES in children undergoing HSCT include hypomagnesaemia, acute GvHD (aGvHD), the use of umbilical cord blood as a stem cell source, the use of granulocyte colony-stimulating factor (G-CSF) and the use of fludarabine as part of the conditioning regimen (68, 69).

The term “posterior reversible encephalopathy syndrome” (PRES) was first coined by Hinchey in 1996 (70) although the syndrome had been described earlier (71, 72). It is a syndrome that usually presents early post HSCT (usually within the first 100 days (73) with a variety of clinical symptoms [seizures, headaches, hypertension, altered mentation, confusion and visual disturbance (74–77) and distinct radiological features (vasogenic oedema of most commonly the parieto-occipital white matter but also the frontal and temporal lobes and posterior fossa] (78–83). The incidence of PRES post HSCT is quite varied in the literature, ranging from 1.6 to 20% (9, 84, 85).

##### Pathophysiology of PRES

The pathophysiology of PRES remains uncertain. There are two main theories regarding the process that leads to the development of vasogenic oedema underlying PRES (86):

1. In the first theory, it is thought that hypertension is the primary trigger. A rapid rise in blood pressure overcomes the autoregulatory mechanisms of the cerebral vessels resulting in hyperperfusion and damage to the capillary bed, causing leakage of fluid into the interstitium.
2. In the second theory, the primary event is speculated to be activation of the endothelium leading to cerebral vasoconstriction and hypoperfusion resulting in vasogenic oedema.

There is more evidence to support the second theory in the literature. Firstly, 20–30% of patients with PRES appear to have normal or mildly elevated blood pressure (87). There are a number of imaging studies which show evidence of hypoperfusion with PRES (88–91). These observations have lead others to hypothesise that the hypertension is a reactive event in an attempt to improve cerebral perfusion and reduce the oedema rather than it being the cause of PRES (86, 92).

##### Clinical Features of PRES

Seizures are the most common presenting feature of PRES in children (68, 93–96). They usually start as non-convulsive focal events and later proceed to convulsive seizures. Non-convulsive status epilepticus has frequently been described (76, 97). Other clinical features include visual disturbance, headache, an altered level of consciousness, nausea and vomiting that may reflect raised intracranial pressure (68, 86, 98, 99).

##### Diagnostic Neuroimaging of PRES

Given the numerous causes of abnormal neurology in the acute post-HSCT setting, a CT scan of the head is often the first choice of neuroimaging. However CT scans are often normal or show non-specific changes in patients with PRES. MRI is the gold standard for PRES diagnosis, with distinctive diagnostic features present in the majority of cases (79). The typical lesions seen are vasogenic oedema in the subcortical and cortical white matter. These are seen as a high signal in T2-weighted images and fluid attenuated inversion recovery (FLAIR) sequences (80). Changes are usually seen bilaterally, most commonly in the parietal and occipital lobes. The frontal and temporal lobes are involved in about half of cases and the cerebellum, brain stem and basal ganglia in about one-third (79, 80, 82, 83). Concurrent intracranial haemorrhage is seen in ~5–19% of patients with PRES (78, 81, 83).

##### Treatment of PRES

The management of PRES in children post HSCT is supportive. It includes the use of antihypertensives, anticonvulsants and withdrawal of the presumed causative agent (68, 98, 100). Anticonvulsant therapy as either primary or secondary prophylaxis is recommended. The duration of therapy varies in the literature from 3–12 months and should be informed by persistence of symptoms, seizures and abnormal electroencephalogram and MRI changes (73, 94, 101, 102). There is little clear guidance in the literature about the use of antihypertensive in PRES, particularly in children. Most papers recommend their use with the caveat that rapid reduction in blood pressure should be avoided to prevent cerebral hypoperfusion. One paper suggests reducing blood pressure by 25% in the first hour and then very gradually in the following hours (103). Electrolyte abnormalities, particularly hypomagnesaemia and bleeding diathesis, should also be corrected (78). In most publications the diagnosis of PRES led to the withdrawal of calcineurin inhibitors and substitution with another immunosuppressant (mostly commonly tacrolimus instead of cyclosporine or vice versa) (9, 68, 99).

Early recognition of PRES is important and the syndrome is usually reversible without long-term sequelae with the supportive treatment described above (68, 99). Although not common, PRES can be life threatening and lead to permanent neurologic sequelae if not treated promptly (104). Straathof et al. demonstrated significantly higher non-relapse mortality in paediatric patients who had cyclosporine-associated neurotoxicity compared to the entire cohort of recipients who had undergone HSCT during the study period at their centre (105). Permanent neurological damage and cerebral infarction in children have also been shown (106). Therefore, prompt recognition and early institution of supportive care and treatment are imperative to ensuring good long-term outcomes.

#### Central Nervous System Graft-Vs.-Host Disease

CNS GvHD as a cause for neurological abnormalities post HSCT is rare and often a diagnosis of exclusion. As neurological manifestations of chronic GvHD (cGvHD) are not included in the National Institutes for Health (NIH)-defined diagnostic criteria (107, 108), they are considered to be “associated with cGvHD,” requiring occurrence together with a manifestation of classic cGvHD in another organ. The diagnostic work-up may include some important considerations: firstly, other causes of CNS neurological abnormalities have to be excluded in a comprehensive diagnostic work-up (as outlined below); secondly, diagnosis of CNS cGvHD may be probable if CNS manifestations are associated with the taper of immunosuppressive treatment (109). Very likely, there is an overlap between CNS cGvHD and autoimmunity, as outlined by Buxbaum and Pavletic (110): as most antibody-driven neurological entities after HSCT manifest in the setting of full donor chimerism, processes of cGvHD and autoimmunity may be assumed and manifestations have been reported such as transverse myelitis, isolated optic neuritis, CNS granulomatous vasculitis, panencephalitis with infiltration of CD3^+^ lymphocytes, and reversible leukoencephalopathy (109–111).

In 2010, a consensus conference of clinical practise in cGvHD, defined CNS GvHD as the presence of the following two mandatory criteria plus at least two facultative criteria (109):

- Mandatory criteria: 1. occurrence of neurological symptoms in the presence of chronic GvHD affecting other organs; and 2. signs of neurological involvement without any other explanation, i.e., no infectious, vascular, or metabolic aetiologies or drug toxicities.
- Facultative criteria: 1. abnormalities on brain MRI; 2. abnormal cerebrospinal fluid (pleocytosis, oligoclonal bands, elevated protein or immunoglobulin G levels); 3. brain biopsy or post-mortem examination revealing GvHD lesions; and 4. response to immunosuppressive therapy.

The consensus conference further defined three types of CNS cGvHD: cerebrovascular disease, demyelinating disease and immune-mediated encephalitis.

Reports of CNS GvHD in the literature are rare. A recently published Frontiers case report and literature review (112) included 46 cases reported between 1990 and 2019. Cases included patients with acute or chronic GvHD prior to or at the time of neurological abnormalities. The median age of onset was 41 years (range 9–68 years) and diagnosis was at a median of 390 days after HSCT (range 7–7300 days). Twenty-five patients had a history of aGvHD, and 29 developed cGvHD prior to or during the onset of neurological symptoms. The clinical characteristics of the 46 patients were variable: 11 presented with stroke-like episodes, 14 had acute demyelinating encephalomyelitis or multiple sclerosis-type manifestations, 17 presented with encephalopathy or encephalitis, and the remaining four had atypical manifestations. The cerebrospinal fluid of 40 patients was tested: 11 of these (27.5%) had no abnormalities. The most common cerebrospinal fluid abnormality was elevated protein, which was present in 23 patients (57.5%). Of the 45 patients who underwent brain MRI, 42 had abnormal findings. The majority of brain biopsies or post-mortem examinations demonstrated immune-mediated changes: perivascular inflammation (*n* = 16, 72.8%), vasculitis (*n* = 4, 18.2%), gliosis, microglia proliferation or activation (*n* = 8, 36.4%), infiltration of CD3^+^/CD4^+^ Tcells (*n* = 1, 4.5%), infiltration of CD3^+^/CD8^+^ T cells (*n* = 6, 27.3%), parenchyma lymphocytic infiltration (*n* = 4, 18.2%),demyelination (*n* = 7, 31.8%), granulomatous infiltration (*n* = 3, 13.6%). Fourty patients received immunosuppressive therapy. Most patients have achieved complete response (*n* = 15) or partial response (*n* = 7) in clinical and/or imaging studies after treatment. Unfortunately, there were inadequate follow-up data to make any conclusions about the outcomes for these patients. Although uncommon, the majority of patients discussed in the review seemed to respond to immunosuppressive therapy.

We were unable to find any definitions of CNS aGvHD in the published literature. In summary, there are clear diagnostic criteria for CNS cGvHD but guidelines regarding CNS aGvHD are warranted.

## Long-Term Neurotoxicity Post HSCT

### Cerebrovascular Accidents

After HSCT, endothelial damage is induced by the conditioning regimen with or without TBI or other types of irradiation and by HSCT complications such as GvHD (113–115). It has been well described that HSCT survivors have a higher prevalence of metabolic syndrome and atherosclerosis, both of which predispose patients to cardiovascular adverse events (including coronary artery disease and peripheral vascular disease), as compared with non-transplanted leukaemia survivors and the general population (114–123). This cardiovascular risk profile predisposes paediatric transplant survivors to myocardial infarction, stroke and peripheral vascular disease. Moreover, it has been well shown that irradiation of the brain may lead to endothelial damage and vasculopathy, which will put HSCT survivors who received TBI conditioning at higher risk of cerebrovascular events (124, 125).

In a report by the American Childhood Cancer Survivor Study (CCSS), in which children with a cancer diagnosis between 1970 and 1986 and who were treated with different disease-specific treatment protocols were included, stroke was reported in 37 childhood leukaemia survivors with a rate of late-occurring stroke of 57.9 per 100,000 person-years [95% confidence interval (CI) 41.2–78.7]. The relative rate (RR) of stroke for leukaemia survivors compared with the sibling comparison group was 6.4 (95% CI 3.0–13.8; *p* < 0.0001) (126). A second CCSS study reported a cumulative incidence of stroke at age 50 years of 6.3% (95% CI 5.1–7.5%) after a median follow-up of 19 years. In comparison, siblings had a cumulative incidence of stroke at age 50 years of 1.1% (95% CI 0.4–1.7%) (127).

Most existing reports on stroke in HSCT survivors included both children and adults (~20% of survivors were <20 years of age at the time of HSCT) and the 10-year cumulative incidence of stroke was 3.5%. Mortality from stroke was 4.0% in HSCT survivors as compared with 1.9% in a population-based comparison group after a median follow-up of 7.0 years (range 2.0–23.7) after HSCT (128, 129). In a European Society for Bone and Marrow Transplantation (EBMT) study, also mainly including survivors who had HSCT in adulthood, the cumulative incidence of a first arterial event 15 years after HSCT was 6% (95% CI 3–10%) (130). There were 20 cardiovascular events of which nine were cerebrovascular accidents across the cohort of 548 patients.

In the studies above, the reported main risk factors for stroke included the presence of components of metabolic syndrome–namely antihypertensive treatment pre-transplant [hazard ratio (HR) 4.8; 95% CI 1.1–21], dyslipidaemia treatment (HR 7.4; 95% CI 1.2–47) (128), a body mass index >30 (HR 3.4; 95% CI 1.1–10.4) (129) – and the presence of ≥2 of the four cardiovascular risk factors hypertension, dyslipidaemia, diabetes, and obesity (RR:12.4; *p* < 0.02) (131). Other risk factors for stroke were disease- or treatment-related and included relapsed disease status (HR 5.9; 95% CI 2.4–14.7) (128) and higher treatment intensity, defined as ≥2 conditioning regimens (HR 8.6; 95% CI 2.9–25.8) and ≥3 conditioning regimens (HR 9.0; 95% CI 2.2–37) (129).

Importantly, neither TBI (as compared with high-dose chemotherapy only conditioning) nor TBI dose or fractionation (less or more than 10 Gy; single fraction vs. multiple fractions) were associated with direct cardiovascular outcomes (129, 131). However, compared with chemotherapy only conditioning, TBI-conditioning and higher TBI dose came out as risk factors for cardiometabolic traits such as the metabolic syndrome and its components (central adiposity, hypertension, insulin resistance and dyslipidaemia) in several studies that followed children after HSCT (118, 132–135). Therefore, with prolonged follow-up, these patients may be at higher-than-expected risk for stroke at older ages and should be longitudinally monitored to ameliorate cardiovascular risk factors where possible.

In summary, after HSCT there appears to be an increased risk for stroke with a cumulative incidence of 3.5–6% after 10–15 years of follow-up, with cardiovascular risk factors such as the metabolic syndrome being the main risk factor. However, as few studies have assessed the long-term risk of stroke in childhood HSCT survivors, the risk for stroke in children transplanted for ALL remains to be determined.

### Secondary CNS Malignancies Post HSCT

Therapeutic improvements over the years have resulted in notably increased chances of survival after myeloablative allogeneic HSCT for paediatric high-risk ALL (136, 137). This increased survival brings a growing population of survivors who are at risk for late therapy-related sequelae. Second malignant neoplasms (SMN) are an unfortunate and distressing complication for childhood HSCT survivors. Large cohort studies have shown that childhood cancer survivors are at 3- to 11-fold increased risk of developing malignancies than the general population, and the incidence increases over time (138–140). Children who have received HSCT form a special risk group within these cohorts (141–144).

Long-running animal studies had already shown that dogs and non-human primates who were given TBI and HSCT developed a significantly higher rate of malignancies than expected after intervals of 1.5 to >20 years (145, 146). When examining a cohort of 7,986 childhood cancer survivors who were treated between 1985 and 2009, Pole et al. observed that children who had received an allogeneic HSCT were at significantly greater risk of developing a SMN than children who had been given an autologous HSCT or had received other treatments for childhood cancer, with cumulative incidences at 15 years of 3.1, 2.5 and 2.3%, respectively; incidence rates diverged more profoundly after ≥15 years (147). A population-based study in 826 adolescents and young adults who had received HSCT for acute myeloid leukaemia (AML), using data from the Centre for International Blood and Marrow Transplant Research (CIBMTR), extrapolated a 10-year cumulative incidence of SMN of 4%, with incidence equally distributed between TBI- and high-dose chemotherapy conditioned patients; 16 non-CNS tumours were diagnosed during a median follow up of 77 months (range 12–194) (148). Chronic GvHD may also be a risk factor for SMN (143, 149). However, this has not been systematically observed in all studies (150, 151), and prolonged immunosuppression may potentially play a role.

Most diagnosed SMNs are solid tumours, among which sit CNS neoplasms. Among the many different reported histologies of second CNS neoplasms, the most often diagnosed neoplasms are meningiomas, low-grade gliomas and high-grade gliomas (150–156), but other pathologies such as ependymomas, medulloblastomas, and supratentorial primitive neuroectodermal tumours occur also (142, 151, 154).

The International Late Effects of Childhood Cancer Guideline Harmonisation Group developed a guideline regarding surveillance for subsequent CNS neoplasms, which was recently published (157). The group concluded that risk of CNS neoplasms was increased after cranial radiotherapy with aggravated risk at higher doses, and that younger treatment age and neurofibromatosis type-1 diagnosis were relevant risk factors. However, they found no high-quality evidence significantly linking exposure to alkylating agents, epipodophyllotoxins, anthracyclines or other chemotherapy to subsequent CNS tumours. They evaluated the sparse evidence linking intrathecal methotrexate and exposure to platinum agents with meningioma development as of small relevance (152, 158). Latency times between primary therapy and development of CNS neoplasms span from 4 to 44.5 years. Cumulative incidence of high-grade gliomas seems to plateau after 14 years, but no such plateau could be established for meningioma incidence. The group did not find sufficient evidence that early detection would reduce morbidity and mortality of CNS secondary neoplasms; therefore, they did not advise routine MRI surveillance for asymptomatic survivors.

Several studies have evaluated the risk of secondary CNS neoplasms after treatment for ALL during childhood. Walter et al. followed 1,612 children treated between 1967 and 1988 for a median of 15.9 years (153). Cumulative incidence at 20 years of CNS neoplasms was 1.39% and of high-grade tumours was 0.7%, with median latency of 12.6 years. Significant risk factors for SMN included presence of CNS leukaemia at diagnosis and use of cranial radiotherapy, with a dose-dependent cumulative risk. These two risk factors were intertwined, as patients with CNS leukaemia at diagnosis were given higher doses of cranial radiotherapy. In a study by Schmiegelow et al., 89% of patients who developed CNS neoplasms after treatment for ALL had received cranial radiotherapy; 5-year survival for non-meningioma CNS neoplasms was dismal (18.3%, standard error ± 3.8%) (154).

With regard to paediatric high-risk ALL patients who undergo HSCT, it can be assumed that it is mainly those patients who receive TBI and/or CNS radiotherapy within their therapy schedules who are at risk for development of secondary CNS neoplasms. This has been confirmed in studies that compared the late effects of TBI conditioning with those of high-dose chemotherapy conditioning in paediatric patients receiving HSCT for leukaemia (144, 150, 156). In adults, some subsequent CNS neoplasms have been described after previous busulfan- and cyclophosphamide-based conditioning for HSCT (159).

Myeloablative TBI-based conditioning regimens used in HSCT, especially in children, have changed over the years from a high-dose single fraction (e.g., 6–10 Gy) to fractionated TBI (e.g., 10–17.5 Gy delivered over multiple days); the most prevalent schedule is now six fractions of 2 Gy given over three consecutive days (160, 161). With regard to secondary neoplasms, including CNS neoplasms, in children and adults, it has been shown that the risk associated with TBI was decreased when the TBI schedule was fractionated, but that this benefit was lost when high total cumulative doses were administered, especially at doses above 14.4 Gy (140, 162). Nevertheless, a British Childhood Cancer Survivor Study population-based study in 17,980 childhood cancer survivors found that even at cranial radiotherapy doses of 0.01–9.99 Gy or 10.00–19.99 Gy (the range in which currently used TBI doses fall), the risk of developing a second CNS neoplasm was already 2-fold and 8-fold increased, respectively, compared with children not receiving cranial radiotherapy (158). When looking at only the risk of subsequent meningioma development, the same pattern holds true (152). Other, often asymptomatic findings on MRI, such as cavernomas, atrophy and white matter abnormalities, can be found in childhood leukaemia survivors and HSCT recipients especially after cranial radiotherapy or TBI (163–165).

Younger age at HSCT also stands out as a risk factor for SMN; children <10 years, especially those <3 years, develop CNS secondary neoplasms at higher rates than older patients, in principal after TBI or cranial radiotherapy (140, 143, 150, 157).

The difficulty of assessing the risk of secondary CNS neoplasms in the paediatric HSCT population is that most studies are performed either in large cohorts including both adults and children or smaller, mostly single centre, paediatric cohorts that do not focus on CNS tumours exclusively. Recently, however, a large multicentre CIBMTR study specifically determined the risk factors for CNS neoplasms after allogeneic HSCT for haematolymphoid diseases in 8,720 paediatric patients between 1976 and 2008, with a case-controlled design, where disease-matched controls had received HSCT but did not develop a CNS neoplasm (151). With 59 CNS tumours developing during follow-up, Gabriel et al. established a 33-times higher than expected rate of CNS neoplasms. The cumulative incidence was 1.29% 20 years after HSCT, and significant risk factors in the entire cohort were having an unrelated donor (HR 3.35, confidence limit 1.77–6.34, *p* = 0.0002) and CNS disease before HSCT for ALL (HR 8.21, confidence limit 2.64–25.56, *p* = 0.0003) or AML (HR 6.21, confidence limit 1.38–28.03, *p* = 0.0174). In contrast, use of TBI, dose of TBI (<12 Gy vs. ≥12 Gy) and age, were not found to be significant risk factors. The lack of significance of TBI as a risk factor can be explained from a statistical point of view: only patients who underwent HSCT for haematologic malignancies were analysed, meaning that the majority of patients in both groups received TBI (88% of patients with CNS tumours and 71% of the controls). Multivariate analysis of the matched patient vs. control cohort (*n* = 168) showed that having an unrelated donor transplant (HR 4.79, confidence limit 1.67–13.78, *p* = 0.0037), CNS disease before HSCT (HR, 7.67, confidence limit 1.78–33.16, *p* = 0.0064), and radiotherapy exposure before conditioning (HR, 3.7, confidence limit 1.19–11.47, *p* = 0.0234) were significant risk factors for SMN. Patients who developed CNS tumours had a 37.2-times higher risk of not surviving compared with the matched controls without CNS tumours (95% CI 26.6–52.0, *p* < 0.0001).The relatively low incidence and long latency between HSCT and development of subsequent CNS neoplasms precludes a recommendation to perform routine MRI surveillance among asymptomatic survivors. However, the devastating consequences of developing a CNS neoplasm, especially those that are malignant, are of such magnitude that HSCT survivors who have received TBI or cranial radiotherapy, their caregivers and healthcare providers should be made aware of the related signs and symptoms, so that appropriate diagnostic actions can be taken when necessary.

### Peripheral Neuropathy Post HSCT

Chemotherapy-induced peripheral neuropathy is a side effect that can interfere with survivors' quality of life even a long time after therapy for childhood ALL (166). Peripheral neuropathy may imply damage to large fibres which is characterised by loss of vibration perception, proprioception and motor control and/or small fibres implying abnormal sensation of heat and cold, paraesthesia, allodynia, spontaneous pain and abnormal perception of thermal stimuli and pain (167).

Following anti-leukaemic therapy, vincristine is historically considered the major cause of peripheral neuropathy (168). In addition, nelarabine as a component of treatment of relapsed T-cell ALL has been associated with severe and sometimes irreversible peripheral neuropathy and pain (169). The additional risk posed by HSCT was recently described in a cross-sectional study of 25 paediatric ALL patients undergoing HSCT. At a median of 8.25 years post HSCT, signs of small and large fibre dysfunction were present in 88 and 68% of patients, respectively, and 50% presented abnormal sensation to pain stimuli (167). In comparison, the same authors found that, in a group of ALL survivors treated with chemotherapy alone, about 66 and 33% of patients had abnormal small and large fibre dysfunction, respectively, and 30% reported abnormal pain sensation at a median 2.5 years post therapy (168).

These studies indicate an additional effect of HSCT on the risk of peripheral neuropathy and pain. This may be due to immune-mediated mechanisms, neuropathies and associated muscle cramps have been described in series of patients with cGvHD (109, 170). Furthermore, immunosuppressive drugs, especially cyclosporine and tacrolimus, may induce peripheral neuropathy (171) and pain syndromes (172, 173) in paediatric HSCT patients.

A study with long-term follow-up of paediatric cancer patients, at a median of 8.5 years after treatment, found peripheral neuropathy to be associated with impaired performance on distal sensory and motor tasks compared with healthy controls and concurrent impact on patients' and parents' reported outcome and quality of life (QoL) (174). Only about 25% of these patients had undergone HSCT. Larger, prospective studies are needed in order to fully evaluate the extent and implications of neuropathy and pain syndromes after paediatric HSCT.

### Long-Term Neurocognitive Outcomes Post HSCT

#### Impact on Cognition

Long-term neurocognitive effects of oncologic treatment for ALL have been recognised since the 1970s. With the understanding that prophylactic CNS therapy could prevent CNS recurrences of ALL, overall survival increased dramatically. In the early protocols, prophylactic CNS-directed therapy consisted of intrathecal methotrexate and cranial radiotherapy to a dose of 24 Gy. In an early prospective study published in 1976 of 23 children undergoing HSCT, 12 months post complete remission 12 children had developed neurologic symptoms, including limping, poor coordination, seizures, ataxia, hyperactivity, and learning disabilities (175). As other studies reported detrimental effects on neurocognitive functioning, especially in children aged <3 to 5 years, cranial radiotherapy doses were decreased to 18 Gy. However, many studies did not find any improvement for cognitive effects, potentially also because of interactive effects of increased doses of methotrexate in many studies (176, 177). A recent mathematical model from the Paediatric Normal Tissue Effects in the Clinic (PENTEC) international consortium calculated the detrimental interaction between cranial radiotherapy and administrated methotrexate with regard to the risk of intelligence quotient (IQ) decrease after treatment and generated dose- and other risk-factor-related normal tissue complication probability models (178). The risk of an IQ <85 was 5% for children who had received a whole-brain dose of radiotherapy of 18.1 Gy, and methotrexate increased any risk of an IQ <85 in equivalence to a generalised uniform brain dose of 5.9 Gy. Because greater event-free survival has been observed in standard-risk ALL patients without prophylactic cranial radiotherapy, the practise is now reserved for selected high-risk CNS3 or CNS relapse cases (179–181). However, even in children treated for ALL without radiotherapy, IQ deficits of 6–8 points and deficits in other domains such as working memory, information processing speed and fine motor functioning as compared with healthy controls are frequent (182, 183).

Within the context of allogeneic HSCT for ALL, some high-risk CNS disease protocols involve cranial radiotherapy or craniospinal irradiation boost before TBI in the conditioning schedule. Hiniker et al. performed TBI to a dose of 12–13.2 Gy in 1.2 Gy fractions with a cranial radiotherapy or craniospinal irradiation boost to a median dose of 24 Gy (range 14–35.4 Gy) in 41 paediatric ALL patients (184). With a median follow-up of 89.7 months, neurocognitive testing revealed a mean post-HSCT overall IQ of 103.7 at 4.4 years. Pre- and post- HSCT neurocognitive testing revealed no significant change in IQ (mean increase +4.7 points). Relative deficiencies in processing speed and/or working memory were noted in six of 16 tested patients (38%). Regarding paediatric leukaemia patients who only received radiotherapy in the form of single-dose or fractionated TBI before HSCT, studies in the 1980s and 1990s reported mostly small but significant decrements in IQ or sensory-motor and cognitive function, although profound effects were observed in children receiving TBI before age 3–4 years (185–187). Kramer et al. found IQ and developmental decline in 65 tested children. Baseline IQ was 110.5 [standard deviation (SD) 14.3] and this fell to 94.5 (SD 16.7) at 1 year after HSCT; 26 patients were re-evaluated at 3 years post HSCT and showed no further changes in IQ (188). However, other researchers did find progressive deficits over >5 years of follow-up in patients with haematologic malignancies treated with HSCT as compared with siblings, especially after previous cranial radiotherapy and/or other CNS prophylaxis (189). Willard et al. concluded that the continuous decline in IQ after HSCT for various diagnoses was only observed in TBI-treated children, as children treated with chemotherapy only conditioning showed recovery in their IQ scores 3 to 5 years after HSCT (190).

In contrast, various other small and large studies found no significant changes in children's neuropsychological or cognitive status after HSCT, even with TBI-based conditioning (15, 191–195). In a study of 158 mixed-diagnosis paediatric patients undergoing HSCT, Phipps et al., found some significant differences in 5-year follow-up graph slopes of IQ and academic achievement measurements, based on diagnosis, type of transplantation, use of TBI, and presence of GvHD (194). However, these differences were small and of limited clinical significance compared with the effect of socioeconomic status of the children on their IQ and academic achievement.

Disparities in outcomes reported by different studies may be partly explained by the different patient populations studied: most study populations consisted of children with various malignant and non-malignant diseases, although the majority usually had acute leukaemia. Therefore, other factors surrounding high-risk ALL patients, such as CNS disease and intensive pre-HSCT (CNS-directed) treatment, may have an important additive effect on core neurocognitive functioning and academic as well as social achievements (183).

One recent study published in 2020 assessed neuropsychological outcomes and anatomical changes on MRI at a median of 5 years after therapy completion in paediatric high-risk ALL patients who were treated with (*n* = 15) or without (*n* = 14) HSCT with fractionated TBI using a protocol that was otherwise similar to the ALL Intensive Chemotherapy Berlin-Frankfurt-Münster (ALL-IC-BFM) 2002 study (163). Outcomes were compared with those of newly diagnosed ALL patients without CNS involvement and hence no disease-related MRI changes (*n* = 16). Compared with non-transplanted patients and pre-treatment controls, patients receiving HSCT had significantly lower volumes of white and grey matter and subcortical structures including the thalamus, hippocampus, putamen, globus pallidus and accumbens. In addition, patients receiving HSCT had generally lower cognitive performance, especially in vocabulary, visuospatial ability, executive functions and attention, and processing speed than other patients. Both treated cohorts performed comparably to controls on all measures related to learning capacity and memory. The thalamus volume was correlated with neuropsychological performance in verbal functions, executive functions and processing speed. There was a general trend for decreased brain volumes in high-risk ALL survivors compared with the pre-treatment controls. This study underlines the added detriment of TBI-based HSCT in high-risk ALL patients, although the relationship between cognitive decline and neuroanatomical changes has been previously described in paediatric ALL patients treated with chemotherapy only (196).

Of course, many biologic and sociodemographic factors influence overall neurocognitive functioning of ALL patients before and after HSCT. Kupst et al. found that pre-HSCT functioning in 153 children with multiple diseases was strongly predictive of later functioning (15). During the course of the disease, children lose developmental and educational opportunities in relation to their peers. Even maternal depression rates can influence children's cognitive tests (197). Moreover, cognitive function does not always directly relate to educational functioning (197). One influential factor that stands out is age at HSCT. The repeated observation that TBI-based conditioning before HSCT results in significantly worse cognitive outcomes for children transplanted before age 3–4 than for older children, is one of the main reasons to refrain from TBI at such young ages (190, 193, 197–200).

It is difficult to compare studies of neurocognitive function with one other. Different study methodologies, patient characteristics, treatment schedules, use or lacking of baseline testing, comparisons with control groups, and the length and manner of follow-up hamper direct comparisons. A major issue is the difference in testing instruments that are applied throughout studies; in cohorts with longer follow-ups, reports can present different outcomes related to changes in test instruments over time (193, 194).

It remains important to remember that, although declines in cognitive function may be measurable for paediatric high-risk ALL patients followed up after HSCT, the vast majority of these children will still display neurocognitive functioning skills within the average population range, and very-long-term neurocognitive quality-of-life effects seem only moderate (201). Notwithstanding this, it is of vital importance that paediatric high-risk ALL patients are monitored and supported from early in their treatment and are followed up, especially after HSCT, in order that they can start required early interventions to negate any decline in neuropsychological, cognitive and academic function as much as possible.

An expert review from the CIBMTR and EBMT on the neurocognitive dysfunction in both adult and paediatric HSCT recipients recommends neurocognitive testing in children before and 1 year after HSCT and then at the beginning of each new stage of education. That review includes a table summarising the validated tests for various neurocognitive domains, the applicable age ranges and time required apply the test (202).

#### Fatigue

Fatigue refers to “the persistent, subjective sense of physical, emotional, and/or cognitive tiredness or exhaustion that is not proportional to recent activity and interferes with usual functioning” (203). The reported prevalence of severe fatigue following paediatric haematological cancers ranges from 1.8 to 35.9% (204). The aetiology is probably multifactorial, representing a complex interaction of chemotherapy- or radiation-induced damage, psycho-social factors, medical comorbidities and immunological/inflammatory mechanisms. In a follow-up study of 76 paediatric patients (of whom 69.7% had received TBI) 5–14 years post HSCT, the mean levels of self-reported and parent-reported fatigue were moderately elevated compared to normative values and were significantly higher than in healthy peers (205). Self-reported fatigue was associated with poorer functioning across all quality of life domains and with more concerns regarding internalising problems, emotional symptoms and personal adjustment (205). Excessive daytime sleepiness–the tendency to doze off or fall asleep in various situations (203)–was reported by 21% of parents and 28% of survivors in the same study.

Randomised trials (206) and longitudinal studies (207) of cancer and HSCT survivors indicate a significant and clinically relevant effect of physical activity on reducing fatigue. A meta-analysis of more alternative mind-and-body practises found a significant positive effect of mindfulness and relaxation practises on fatigue in primarily adult cancer patients, while acupuncture, massage and energy therapy showed no significant effect (208). These results, which need to be confirmed, might indicate that practises aimed to reduce fatigue where the patient takes an active role in execution and in symptom management have a higher success rate than practises where individuals take a more passive role and are reliant on practitioners to administer therapies.

Although prospective long-term studies are needed to fully assess the extent and severity of fatigue following HSCT for childhood leukaemia, both the suspected incidence and the possibilities for effective interventions indicate that screening for fatigue and excessive tiredness should be a priority in long-term follow-up consultations after allogeneic HSCT.

#### Quality of Life

With increasingly better outcomes following childhood cancer, including ALL, health-related quality of life (HRQoL) has become an important outcome measure for paediatric oncology that might guide clinical decisions in cases where different protocols have the same survival outcomes (209). Studies of HRQoL in paediatric HSCT recipients often have differences in design, follow-up time, heterogeneity in diagnoses, treatment regimen and scoring instruments (210). Instruments to validate paediatric QoL scoring systems specific to the HSCT population are under development (211).

HSCT recipients are at high risk of late effects (212–216); a recent study on survivors of haematological malignancies found that 47% of HSCT recipients and 22% of patients who received only chemotherapy suffered from multiple chronic health conditions 10–33 years post diagnosis (215). The presence of a chronic health conditions is the strongest predictor of reduced HRQoL in leukaemia survivors (212, 214–216). Indeed, the number of chronic health conditions seems to have a greater impact on the long-term HRQoL than the treatment modality the patient received (HSCT vs. chemotherapy only) (215). However, the inclusion of TBI in the conditioning regimen has been associated with impaired psychosocial functioning beyond the first year post HSCT (217).

The presence of chronic GvHD and related chronic health conditions impact on patients' quality of life both early (within 2 years) (218) and in the long term (>10 years) (213). Chronic pain (174, 213, 214), anxiety (214, 215) and fatigue (205, 215) seem to negatively influence long-term quality of life. However, the extent and severity of these problems needs to be further investigated in longitudinal studies.

Using comparisons against healthy controls, studies from the last decade of quality of life post HSCT have primarily observed differences in the physical components of HRQoL scores (214, 215, 218). However, the results are not uniform. Berbis et al. found that patients who underwent HSCT had lower HRQoL than population norms for all QoL domains except physical composite scores, bodily pain and general mental health (213). Visentin et al. found both physical and mental composites scores to be decreased at a mean of 7.6 years post HSCT compared to age-and-sex-matched French reference scores (219). A very recent study by Yen et al. reported no difference in mental component summary scores but significantly higher levels of anxiety, fatigue, sensation abnormalities and memory problems in HSCT recipients 11–28 years post treatment compared with non-cancer controls (215). Lastly, Sundberg et al. found that being unemployed or on sick leave was a stronger predictor of reduced quality of life than HSCT in long-term (>10 year) survivors of lymphoblastic malignancies, underlining the importance of including measures of social and societal functioning in research and follow-up consultations (220).

Several studies indicate a positive effect of physical training on HRQoL for childhood leukaemia survivors (217, 221), although the timing and optimal modality of this training has not been uniformly defined (222). A recent study by Davis et al. on 20 HSCT recipients who received TBI based conditioning. found that even at a mean of 8.4 years post HSCT (range 2.3–16 years) a 6-month supervised exercise intervention significantly improved physical health, emotional, social and school domains as well as overall quality of life compared to pre-intervention (223). The improvement was maintained at 6 months after the intervention, suggesting a role for physical rehabilitation even at long-term follow-up clinics post HSCT.

## Immune Effector Cell Associated Neurotoxicity Syndrome

Neurological toxicity has been described in virtually every trial using CAR T cell therapy for haematological malignancies (224). Following the initial descriptions of the neurotoxity associated with CAR T cells, it was initially speculated that the Fludarabine, used for lymphodepletion, may have been responsible (225). However with more time, experience and the use of alternative lymphodepletion regimens, it has become clear that the timing and neurological symptoms are distinct from those seen with fludarabine toxicity and that Fludarabine is not primarily responsible for CAR T cell associated neurological toxicity (226).

At the time of writing this paper Tisagenlecleucel (Kymriah, Novartis), an autologous CD19-CAR Tcell, is approved by the US Food and Drug administration (FDA) and other governmental bodies for use in children and young adults for relapsed or refractory CD19+ B ALL, including post HSCT relapse. The pivotal phase 2 study (ELIANA trial) administered Tisagnelecleucel to 75 children and young adults with relapsed and refractory CD19+ B ALL (227). Neurological events occurred in 40% of patients within 8 weeks of infusion. Grade 3 neurological events occurred in 13% and there were no grade 4 neurotoxicity and no reported cerebral oedema. Clinical presentation included encephalopathy, confusion, delirium, tremor, agitation, somnolence and seizures. Neurological events usually occurred at the same time as cytokine release syndrome (CRS) or shortly after it's resolution. Median time to develop ICANs was 8 days and the median duration of symptoms was 7 days. Severe neurological events occurred more frequently in patients with severe CRS.

Reassuringly, real world data published recently using data provided to the CIBMTR on 255 children and young adults (median age 13.2 years) who received Tisagenlecleucel for relapsed or refractory CD 19+ ALL showed lower rates of ICANs than the ELIANA trial (228). The incidence of any neurological event was 27.1% (and 9% for ≥grade3 ICANs). The time to develop symptoms and duration of ICANs were similar with that seen in the ELIANA trial. The most common symptoms were reduced consciousness (47.8%), tremors (21.7%), seizure (18.8%), hallucinations (17.4%) and dysphasia/aphasia (15.9%).

The most frequently identified risk factors for the development of ICANS are disease burden and peak CAR T cell expansion (225, 229–232). Other risk factors include extramedullary disease (229, 231), younger age, pre-existing neurological abnormalities, higher CAR T cell dose and cytopenias (225, 231) and high grade CRS (227).

The recently published clinical practise guideline for immune effector cell related adverse events from the Society for immunotherapy for cancer (SITC) (231) provides clear guidance for the grading, investigation (pre, during and post) and management of ICANs following CAR T cell therapy.

There is nothing yet in the literature about the long term CNS complications of CAR T cell therapy.

## Conclusion

In this review, we have provided a comprehensive review of both the acute and long-term neurological complications in children following HSCT for ALL. The majority of the literature on acute neurotoxicity is in the adult population, although some studies included children and a minority of studies focussed on paediatric HSCT recipients (1–6, 104, 233, 234). Within these studies, the paediatric populations were heterogeneous, with children undergoing HSCT for a variety of indications and use of different stem cell sources and a range of conditioning regimens. Possibly due to this heterogeneity, the reported incidence of acute neurotoxicity varies widely from 10 to 57% (1–6), but overall appears high. What is clear is that acute neurological complications are associated with significant mortality, with mortality rates of up to 10% reported (1, 4, 6, 104).

Identified risk factors for CNS complications include aGvHD, alternate donors and the use of TBI-based conditioning regimens (1, 4, 6, 104). TBI being a risk factor for neurological complications is an important consideration for the approach to HSCT in paediatric ALL. However, the recently published For Omitting Radiation Under Majority Age (FORUM) study has clearly identified that TBI-based conditioning regimens provide a survival advantage for children ≥4 years. Therefore, for the foreseeable future TBI will continue to be used for the majority of children with ALL undergoing HSCT (137).

The major acute CNS toxicities in children post HSCT relate to infections and drug-related toxicities (from conditioning agents and GvHD prophylaxis). The majority of reviews focussing on paediatric HSCT recipients concentrate on short-term CNS complications. We chose to include long-term neurotoxicity in this review, specifically cerebrovascular accidents, SMNs, peripheral neuropathy and neurocognitive outcomes (including cognition, fatigue and quality of life). We believe it is essential to improve our understanding of long-term neurological complications of HSCT as more children undergoing this treatment are becoming long-term survivors. This is particularly relevant as long-term neurological toxicities can significantly impact on the quality of life for survivors.

How acute neurotoxicities such as CNS infections and drug toxicities impact on long-term outcomes–especially neurodevelopmental, neurocognitive and quality of life outcomes–is understudied and largely unknown. As an example, how viral- or drug-associated encephalopathy, which usually occurs as an acute complication of HSCT, impacts on long term neurocognitive outcomes is not clear. In addition, whether children who develop an acute neurotoxicity are at greater risk of developing a long-term neurological complication is not known. As more children are expected to become survivors of HSCT for ALL, it is important to understand how the acute toxicities can affect the developing brain in the long term: this should be a priority for future studies.

The impact of acute neurological complications on long-term outcomes is particularly important to understand in the current era, with the advent of CAR T-cell therapy for ALL. Immune effector cell-associated neurotoxicity syndrome (ICANS) is a well-recognised early complication of CD19-targeted CAR T-cell therapy for patients with relapsed ALL when used before or after HSCT. The incidence of ICANS was 40% in the ELIANA trial of the CD19-targeted CAR T-cell therapy tisagenlecleucel for children and young adults with pre-B-cell ALL (227). The long-term CNS complications of CAR T cell therapy are not yet known and is an important area for research as children become long-term survivors of this type of therapy.

In conclusion, the exact risk assessment of developing neurotoxicity for an individual patient undergoing HSCT for paediatric ALL is difficult due to the lack of good studies in this area. The risk of acute neurological symptoms such as seizures or encephalopathy (PRES, infections, Busulfan) and peripheral neuropathy (Vincristine, Calcineurin inhibitors) are relatively high with estimates at 5–10% (18–20, 45, 47, 48, 68, 76, 93, 104) and 10–50% (167, 170–172), respectively and should lead to specific considerations during the pre-HSCT assessment and the informed consent process with families prior to HSCT. Furthermore, the risk of more durable or late occurring neurotoxicity such as stroke or secondary brain tumours is higher than background population at an estimated risk of at least 2–4 times higher, probably rather 4–8 times higher (128–130, 151, 158). Cognitive impairment following TBI may be less pronounced with modern HCT modalities, but risk of fatigue in the early post-transplant years (205) and risk of reduced brain processing speed may be relevant. Memory, attention and changes in IQ has not yet been shown to be significantly impacted (184–195). However, this may change as the population of paediatric HSCT survivors gets older. More research is needed for both the acute and long-term neurological complications in children undergoing HSCT for ALL.

## Author Contributions

All authors listed have made a substantial, direct, and intellectual contribution to the work and approved it for publication.

## Conflict of Interest

The authors declare that the research was conducted in the absence of any commercial or financial relationships that could be construed as a potential conflict of interest. The Reviewer GL declared a past collaboration with several of the authors, AL and MI, to the handling editor. The handling editor declared a shared consortium with the authors at time of review.

## Publisher's Note

All claims expressed in this article are solely those of the authors and do not necessarily represent those of their affiliated organizations, or those of the publisher, the editors and the reviewers. Any product that may be evaluated in this article, or claim that may be made by its manufacturer, is not guaranteed or endorsed by the publisher.
